# Earthworm Effects without Earthworms: Inoculation of Raw Organic Matter with Worm-Worked Substrates Alters Microbial Community Functioning

**DOI:** 10.1371/journal.pone.0016354

**Published:** 2011-01-27

**Authors:** Manuel Aira, Jorge Domínguez

**Affiliations:** Departamento de Ecoloxía e Bioloxía Animal, Facultade de Bioloxía, Universidade de Vigo, Vigo, Spain; Duke University, United States of America

## Abstract

**Background:**

Earthworms are key organisms in organic matter decomposition because of the interactions they establish with soil microorganisms. They enhance decomposition rates through the joint action of direct effects (i.e. effects due to direct earthworm activity such as digestion, burrowing, etc) and indirect effects (i.e. effects derived from earthworm activities such as cast ageing). Here we test whether indirect earthworm effects affect microbial community functioning in the substrate, as when earthworms are present (i. e., direct effects).

**Methodology/Principal Findings:**

To address these questions we inoculated fresh organic matter (pig manure) with worm-worked substrates (vermicompost) produced by three different earthworm species. Two doses of each vermicompost were used (2.5 and 10%). We hypothesized that the presence of worm-worked material in the fresh organic matter will result in an inoculum of different microorganisms and nutrients. This inoculum should interact with microbial communities in fresh organic matter, thus promoting modifications similar to those found when earthworms are present. Inoculation of worm-worked substrates provoked significant increases in microbial biomass and enzyme activities (β-glucosidase, cellulase, phosphatase and protease). These indirect effects were similar to, although lower than, those obtained in pig manure with earthworms (direct and indirect earthworm effects). In general, the effects were not dose-dependent, suggesting the existence of a threshold at which they were triggered.

**Conclusion/Significance:**

Our data reveal that the relationships between earthworms and microorganisms are far from being understood, and suggest the existence of several positive feedbacks during earthworm activity as a result of the interactions between direct and indirect effects, since their combination produces stronger modifications to microbial biomass and enzyme activity.

## Introduction

Decomposition is one of the key processes that regulates nutrient cycling in terrestrial ecosystems, which is mainly carried out by microorganisms, although decomposer food-webs are highly complex and include protozoa, nematodes, microarthropods and earthworms, among others [1]–[3]. When present, earthworms play a critical role in the decomposition of organic matter, significantly accelerating decomposition rates and nutrient turnover [4], [5]. Earthworms directly affect the decomposition of soil by modifying the organic matter and microorganisms that pass through the earthworms’ guts, which are released via casting, namely gut associated processes [6], [7]. In this way, microbial community structure is significantly affected by passage through the earthworm gut, an effect that also depends on the earthworm's diet [8]–[10]. Moreover, microorganisms also flourish in earthworm galleries because of the presence of mucus and casts [11], and because of physical changes in the substrate that favour aeration. All these are direct effects, defined here as the effects on microbial communities caused by the joint action of earthworm cast production and gallery formation (and other processes such as mucus production); these effects are density-dependent [12]. Decomposition pathways also include the effects caused by microbial communities on decaying organic matter, which modify both nutrient contents and the composition of their communities through ecological succession [13]. Changes in microbial communities due to the presence of earthworm-worked material into unworked material may also alter this decomposition pathway indirectly, possibly by modifying the composition of the microbial communities involved in decomposition, thus probably affecting the decomposition rates [12]. Furthermore, the contact between worm-worked and unworked material may influence the decomposition process, as well as how the worm-worked material undergoes natural ageing processes, which generally lead to a continuous decline in microbial biomass and activity, and to changes in the bacterial/fungal ratio [14], [15] Contact between different processed materials thus involves the transportation of microorganisms between substrates that are decomposed to diverse degrees (due to the movement of earthworms) as well as activation of specific subsets of microbial communities via the increased availability of nutrients. Indirect effects are derived from direct effects, and include processes such as the ageing of earthworm-worked material and mixing of such material with substrates that have still not been processed by earthworms. According to this rationale, it is difficult to separate direct and indirect processes and their components, because they occur simultaneously in time and space.

Epigeic earthworms are litter dwellers and in soils inhabit and transform fresh organic matter contained in forest litter, litter mounds and herbivore dungs, as well as man-made environments such as manure heaps. These habitats, soils and waste heaps, are hotspots of heterotrophic activity, in which epigeic earthworms interact intensively with microorganisms and other organisms within the decomposer community, thus strongly affecting decomposition processes [2], [12], [16]. In these habitats earthworms feed on substrates that due to their particle size and composition are easy to ingest and assimilate, producing holorganic casts that are difficult to separate from the ingested substrate. Epigeic earthworms may affect decomposition through ingestion, digestion, assimilation in the gut and then casting (direct effects); and cast associated processes (indirect effects), which are more closely related to ageing processes, the presence of unworked material and to physical modification of the egested material (weeks to months). Burrowing activities lead to physical modifications of the substrate, such as aeration and leaching [17], and are expected to be more important in soil-dwelling than in epigeic earthworms. Vermicomposting is an example of an enhanced decomposition process, in which the joint action of earthworms and microorganisms decompose and stabilize raw materials [18]. Thus, vermicomposts contain microbial communities that differ from those in the parent material [19]. Although the changes that take place depend greatly on the earthworm species, different types of earthworms create similar microbial community structures in vermicomposts produced from different starting materials [20]. Moreover, vermicompost like other earthworm-worked material undergoes ageing processes that are associated with decreased microbial biomass and enzymatic activity [21].

Here we analyse whether and to what extent the indirect effects of earthworms are able to alter the microbial properties of fresh organic matter, thus modifying decomposition rates, independently of the presence of earthworms. Furthermore, we question whether these indirect effects also depend on the earthworm species, since it is known that earthworm-induced modifications to the microbial and nutrient content of substrates that they ingest greatly depend on the species of earthworm involved [20]. We also study whether indirect effects are density-dependent, as previously documented for direct effects [12], or if the response is independent to the amount of worm-worked material present (microorganisms). To address these questions we inoculated fresh organic matter (pig manure) with worm-worked substrates (vermicompost) produced by three different earthworm species. Two doses of each vermicompost were used (2.5 and 10%). We monitored the response of microbial biomass and activity and changes in four enzymatic activities associated with the C, P and N cycles (β-glucosidase, cellulase, alkaline phosphatase and protease). We hypothesized that the presence of worm-worked material in the fresh organic matter will result in an inoculum of different microorganisms and nutrients. This inoculum should interact with microbial communities in fresh organic matter, thus promoting modifications similar to those found when earthworms are present.

## Results

**Figure 1:**
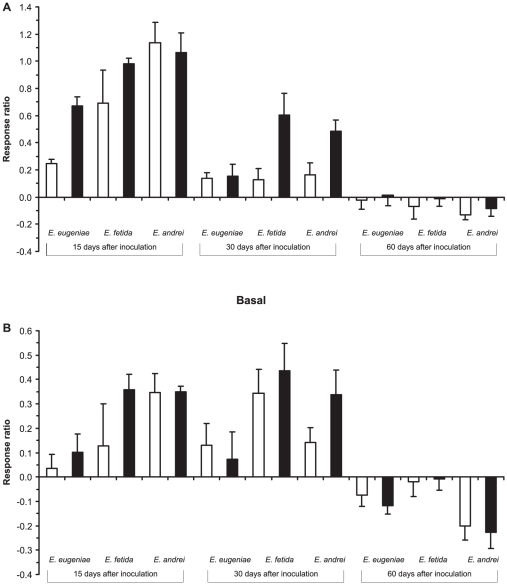
Effects of type of vermicompost, dose of inoculation and time on microbial biomass and activity. Shown are the changes in the a) substrate induced respiration and b) basal respiration in presence of inoculum of vermicompost (2.5 and 10%) relative to control (no inoculum) calculated as (V_i_-V_c_)/V_c_, where V_i_ and V_c_ are the values of the variables of samples inoculated with vermicompost or controls. White and black bars represent 2.5 and 10% of inoculation respectively. Mean ± SE (n = 5).

**Figure 2:**
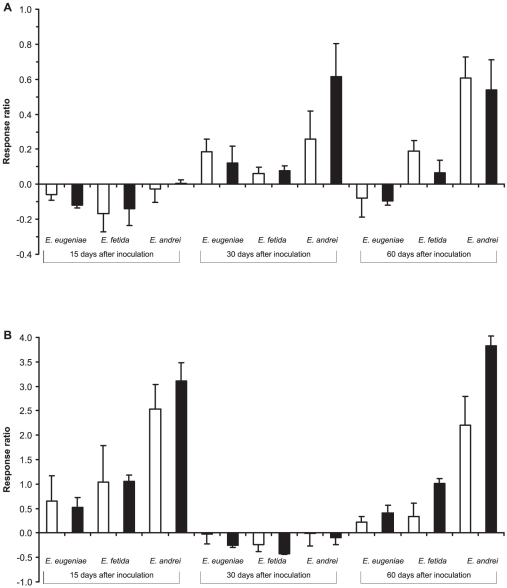
Effects of type of vermicompost, dose of inoculation and time on C metabolism. Shown are the changes in the a) β-glucosidase activity and b) cellulase activity in presence of inoculum of vermicompost (2.5 and 10%) relative to control (no inoculum) calculated as (V_i_-V_c_)/V_c_, where V_i_ and V_c_ are the values of the variables of samples inoculated with vermicompost or controls. White and black bars represent 2.5 and 10% of inoculation respectively. Mean ± SE (n = 5).

The inoculation of earthworm-worked substrates significantly affected the microbial community in pig manure over time. The microbial biomass, measured as substrate-induced respiration (SIR), clearly increased with the dose of inoculation (F_1,72_ = 11.92, P = 0.0006; Fig. 1a), and in samples inoculated with 10% vermicompost the values were almost twice as high in samples inoculated with 2.5% vermicompost. Furthermore, the SIR strongly depended on type of vermicompost. Thus, and in the samples SIR in pig manure inoculated with vermicompost produced by *E. fetida* and *E. andrei*, was the double than pig manure inoculated with vermicompost produced by *E. eugeniae* (Fig. 2a). Interestingly, this effect was only evident 15 days after inoculation, and disappeared after 30 and 60 days, resulting in a significant interaction between type of vermicompost and time (F_4,72_ = 6.23, P = 0.0002; Fig. 2a). On the contrary, the basal respiration was maintained 30 days after inoculation, finally falling below that in the non-inoculated samples after 60 days (F_2,72_ = 34.05, P<0.00001; Fig. 1b). The type of vermicompost also had a significant effect on basal respiration (F_2,72_ = 7.35, P = 0.001; Fig. 1b). Basal respiration in samples inoculated with vermicomposts produced by *E. fetida* and *E. andrei* was higher (10 and 6 times respectively) than in samples inoculated with vermicompost produced by *E. eugeniae*. However, the dynamics of decomposition differed in the three vermicomposts (type of vermicomposting x time interaction: F_4,72_ = 3.85, P = 0.0068; Fig. 1b), microbial activity peaked after 30 days in samples inoculated with *E. fetida*, but microbial activity decreased continuously in samples inoculated with *E. andrei* vermicompost) or was maintained after 30 days, with a final decrease in samples inoculated with *E. eugeniae* vermicompost.

Inoculation of pig manure with worm-worked substrates also modified the activity patterns of the four enzymes analyzed. Thus the β-glucosidase activity was strongly affected by the type of vermicompost (F_2,72_ = 23.08, P<0.00001; Fig. 2a), samples inoculated with vermicompost produced by *E. andrei* this activity were up to 22 and 42 times higher than in samples inoculated with vermicomposts produced by *E. fetida* and *E. eugeniae* (Fig. 2a). Overall β-glucosidase activity increased over time (F_2,72_ = 18.67, P<0.00001), especially in samples inoculated with *E. fetida* and *E. andrei* vermicomposts, samples inoculated with vermicompost produced by *E. eugeniae*, the enzyme activity decreased after 60 days, resulting in an interaction between type of vermicompost and time (F_4,72_ = 4.89, P = 0.0015; Fig. 2a).

Cellulase activity was also strongly affected by the type of vermicompost inoculated (F_2,72_ = 45.85, P<0.0001; Fig. 2b), cellulase activity in samples inoculated with vermicompost produced by *E. andrei* was up to 4 and 7 times higher than activity in samples inoculated with the other vermicomposts. Cellulase activity also differed over time, with a marked decrease after 30 days (F_2,72_ = 46.23, P<0.0001), although the effect depended on type of vermicompost, owing to the marked increase at 60 days in samples inoculated with vermicompost produced by *E. andrei* (type of vermicompost x time interaction, F_4,72_ = 9.89, P<0.0001). Moreover, patterns of cellulase activity differed between dose of inoculation (dose of inoculation x time interaction; Fig. 2b, F_2,72_ 37.05, P<0.05), with an increase in activity in samples inoculated with 10% of vermicompost after 30 and 60 days, but not in samples inoculated with 2.5% vermicompost.

**Figure 3:**
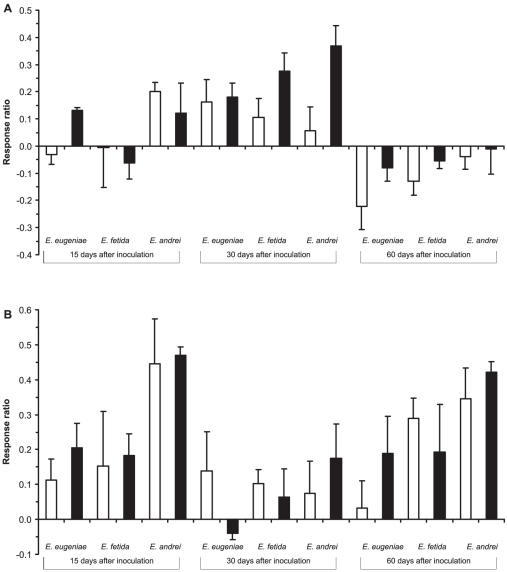
Effects of type of vermicompost, dose of inoculation and time on P and N metabolism. Shown are the changes in the a) alkaline phosphatase and b) protease activity in presence of inoculum of vermicompost (2.5 and 10%) relative to control (no inoculum) calculated as (V_i_-V_c_)/V_c_, where V_i_ and V_c_ are the values of the variables of samples inoculated with vermicompost or controls. White and black bars represent 2.5 and 10% of inoculation respectively. Mean ± SE (n = 5).

The type of vermicompost inoculated affected the alkaline phosphatase activity (F_2,72_ = 3.33, P = 0.0415; Fig. 3a), samples inoculated with vermicompost produced by *E. andrei* showed five times higher activity than samples inoculated with the other vermicomposts. Furthermore, the activity in samples inoculated with 10% vermicompost was 8 times higher than in samples inoculated with 2.5% vermicompost (F_1,72_ = 6.18, P = 0.0152). The pattern of alkaline phosphatase activity differed significantly over time, with an increase after 30 days followed by a decrease after 60 days (F_2,72_ = 22.35, P<0.0001).

Protease activity depended on the type of vermicompost used (F_1,72_ = 9.17, P<0.0002; Fig. 3b), pig manure inoculated with vermicompost produced by *E. andrei* showed 3 times higher activity than in samples inoculated with the other two vermicomposts. The pattern of activity over time was similar to that of cellulase activity, with a decrease after 30 days followed by an increase after 60 days (F_2,72_ = 6.92, P<0.0017; Fig. 3b).

## Discussion

We found that the inoculation of vermicompost modified microbial community functioning (i.e. microbial biomass and enzyme activities) during decomposition of the pig manure, showing the existence of indirect effects of earthworms. The data reveal that inoculation with vermicomposts had a strong effect on microbial biomass and activity, an effect that mainly depended on the type of vermicompost used. Furthermore, our results clearly indicate that the indirect effects of earthworms on organic matter decomposition are independent of the amount of earthworm-worked substrate inoculated (except for microbial biomass and alkaline phosphatase), suggesting that indirect earthworm effects have a threshold at which functioning is triggered, unlike what was found for direct earthworm effects [12].

Although the vermicompost produced by *E. andrei* had the highest microbial biomass, similar contents were found in samples inoculated with vermicompost produced by *E. andrei* and *E. fetida*. The general dose-dependent increase in microbial biomass (without interaction between type of vermicompost and dose of inoculation) suggests priming effects [22], [23]. In fact, priming effects increase with the amount of organic substances added [22]. Nevertheless, the vermicompost only differed in the nitrate (*E. andrei* > *E. fetida*  =  *E. eugeniae*) and dissolved organic contents (*E. fetida* < *E. andrei*  =  *E. eugeniae*), and microbial biomass in inoculated samples should have responded differently to the type of vermicompost. Hence, we cannot rule out possible effects of interactions between nutrients and microorganisms in parent and inoculated materials since we inoculated a mixture of microorganisms and nutrients, and not only nutrients (priming effects *sensu stricto*, 22). Increases in microbial biomass and activity were also found in the early stages of decomposition of pig manure, although values of microbial biomass and activity were markedly higher when earthworms were present [24]-[27]. However, these effects were not consistent over time (as occurred when earthworms were present), which may be attributed to the fact that microbial biomass and activity decrease rapidly in vermicomposts [21]. Such decreases have been observed during ageing of fresh vermicomposts similar to those used here [21].

Here we use the activity of enzymes as proxy of microbial community functioning and also of the physiological capabilities of microbial communities [28]. The enzymes analyzed responded to the addition of vermicompost suggesting that the presence of vermicompost strongly affected microbial community functioning. The peaks in enzyme activity after 15 days (β-glucosidase) were similar to those found in a previous study with pig manure processed by *E. fetida* [26], [27], although the presence of earthworms produced greater stimulation of the enzymatic activities. The activity of this enzyme increased with the amount of enzyme already present in the vermicompost (*E. eugeniae* > *E. andrei*  =  *E. fetida*), that is, depended on the type of vermicompost. In contrast, the cellulase activity depended on the type of vermicompost used, which did not differ in their cellulase activity (Table 1); however, the activity of the two enzymes was not dose-dependent. These results suggest that not only the amount of enzymes already present in the vermicompost, but also other factors such as the difference in microbial communities, are involved in the observed response to the addition of vermicompost, or the interaction between microbial communities in vermicompost and manure [20], [26].

**Table 1:** Microbiological characteristics of the fresh pig manure and vermicomposts from three earthworm species (*Eudrilus eugeniae*. *Eisenia fetida* and *Eisenia andrei*).

	Pig manure	*E. eugeniae*		*E. fetida*		*E. andrei*	
		Value	Ratio	Value	Ratio	Value	Ratio
Substrate induced respiration (µg CO_2_ g^−1^ om^a^ h^−1^)	620±30^a^	570±20^b^	−0.07^A^	540±20^b^	−0.12^A^	820±10^c^	0.34^B^
Basal respiration (µg CO_2_ g^−1^ om h^−1^)	1100±50^a^	190±20^b^	−0.82	220±40^b^	−0.79	320±30^b^	−0.70
β-glucosidase activity (µg PNP g^−1^ dw)	1200±120^a^	1150±80^a^	−0.03^A^	520±60^b^	−0.56^B^	800±60^b^	−0.32^C^
Cellulase activity (µg eq. glucose g^−1^ dw)	8600±1140^a^	15200±720^b^	0.76^A^	13700±460^bc^	0.59^AB^	18000±1100^bd^	1.09^AC^
Protease activity (µg tirosina g^−1^ dw)	16500±650^a^	7460±860^b^	−0.54^A^	3770±160^c^	−0.77^B^	7020±550^bd^	−0.57^A^
Alkaline phospatase activity (µg PNP g^-1^ dw)	4680±350^a^	5640±390^ab^	0.20^A^	3720±260^ac^	−0.20^B^	5500±250^ab^	0.17^A^

The response ratio (Ratio) was calculated as (value of vermicompost- mean value of control)/mean value of control. Means (n = 5) were separated using Tukey HSD test (p<0.05) after a significant two one-way ANOVA tests (one to compare pig manure and the three vermicompost, another to compare response ratio between the three vermicomposts). Different letters indicate statistical differences; thus, lower case letter compare pig manure with the vermicomposts (value) and capital case letter compare the response ratio (Ratio) between the vermicomposts).

a om. organic matter.

Alkaline phosphatase peaked over time and was highest in samples inoculated with vermicompost produced by *E. andrei*, although enzymatic activity was the same as in the vermicompost produced by *E. eugeniae* (Table 1). Thus the alkaline phosphatase activity strongly depended on dose of inoculation (10%>2.5%), and at least after 15 days, the response to inoculation was that expected from the values of enzyme activity in the vermicompost (Table 1). The activity of this enzyme is regulated by the concentration of orthophosphates (the end-product) [29]. In addition, increases in available phosphorus have been reported, as in vermicompost produced by *E. fetida* independent of the substrate used [30]. Therefore, the inoculation would provide a certain amount of phosphorus, which would reduce the phosphatase activity; however, phosphatase activity was also lower in samples inoculated with 2.5% vermicompost than in those inoculated with 10% vermicompost. Nevertheless, the progressive increase in phosphatase activity over time may indicate that accumulation of phosphorus is not sufficient to stop the enzyme. A similar increase in phosphatase activity has been reported in the presence of earthworms, especially *E. fetida* fed with pig manure, although to a greater extent and only in the first stages (after 7 weeks of vermicomposting) [26], which again suggests that the indirect presence of earthworms is able to alter the dynamics of microbial activity during the decomposition of pig manure.

The increase in protease activity contrasts with the decrease observed after 60 days in the presence of *E. fetida* fed with pig manure [27]. In addition, the highest activity was always recorded in samples inoculated with vermicompost produced by *E. andrei*, even though the enzymatic activity of vermicompost produced by *E. eugeniae* was the same (Table 1). The slight decrease in protease activity over time is consistent with the pattern observed during ageing of vermicompost. The decrease in enzyme activity during ageing was explained by a decrease in microbial biomass followed by degradation of the remaining enzymes [27]. We found a marked decrease in microbial biomass in inoculated samples, but the activity of enzymes (except phosphatase) was maintained throughout the experiment, so that the pool of extracellular enzymes produced by the remaining microorganisms was more active in the manure than in the vermicompost alone. The data contrast with the expected decrease due to indirect effects of earthworms; this may be partly explained by the presence of sufficient substrate in pig manure for the microorganisms from the vermicompost to utilize, without competing with earthworms, as in soils [31]. In fact, despite earthworms may digest microorganisms, we found increases in microbial biomass in pig manure processed by E. fetida [25]–[27] as well as high protease activity in the gut of the same earthworm species [32].

Earthworms strongly improve decomposition rates by modifying microbial composition, and increasing microbial enzymatic activity by enhancing microbial biomass and nutrient release [27]. We found that inoculation of raw organic matter with a worm-worked material produced the same effects, altering microbial community levels of activity, which is a key factor for organic matter decomposition. Furthermore, this process is independent of the dose of inoculation and includes the presence of and interaction between microbial communities from earthworm-worked material and those of un-worked material; these communities are metabolically more diverse and also have higher levels of microbial activity than those in the unworked material [26], [27], thus enhancing decomposition rates. The strength of the process we found greatly depends on the earthworm species. Such earthworm-specific effects are consistent with recent results indicating that different groups of microorganisms are stimulated differently during gut transit, depending on the earthworm species [33], [34], also with differences in gut microbiota related to the earthworm diet [8] and the fact that the microbial community structure of vermicompost is largely determined by earthworm species rather than the parent material [20]. Nevertheless, this effect appears to be transient in microbial communities, since biomass and activity tend to decrease over time, unlike the activities of enzymes that remain associated with humic compounds [35]. Our data shows that the relationship between earthworms and microorganisms are far from being understood, and suggests the existence of several positive feedbacks during earthworm activity as a result of the interactions between direct and indirect effects, since the combined effects lead to greater modifications in microbial biomass and enzyme activity.

## Materials and Methods

### Pig manure and vermicompost

Fresh pig manure was obtained from a pig-breeding farm near the University of Vigo (NW Spain). The pig manure was homogenized in the manure pit, then stored in sealed plastic containers and kept at 5°C until use. Three different vermicomposts derived from from pig manure were obtained from laboratory cultures of the earthworm species *Eudrilus eugeniae*, *Eisenia fetida* and *Eisenia andrei*. Some parameters of microbial biomass, activity and enzyme activities of pig manure and the three vermicompost used are given in Table 1.

### Experiment set-up

We used 200 ml plastic containers (microcosms) covered with perforated lids to hold the mixtures of vermicompost and pig manure. We inoculated the pig manure with two doses (2.5 and 10% per mass basis) of each of the three vermicomposts (*E. andrei*, *E. fetida* and *E. eugeniae*) by carefully mixing the vermicompost with the pig manure until a homogeneous mixture was obtained. The controls (0% vermicompost) were homogenized in the same way; all mixtures had the same weight (100 g fresh weight). We set up five replicates per treatment, i.e. type of vermicompost, rate of inoculation and time (N = 135). Microcosms were maintained under laboratory conditions (20°C) in a scientific incubator. A subset of microcosms for each type of vermicompost and rate of inoculation (n = 5) was destructively sampled after 15, 30 and 60 days.

### Analytical procedures

Microbial biomass and activity were assessed by measuring the rate of emission of CO_2_ from the sample after 12 and 6 hours incubation for substrate induced respiration (SIR) and basal respiration, respectively. Analysis of SIR has been shown to be a good way of measuring microbial biomass in vermicomposting systems [36]. Prior to incubation, 0.75 ml of glucose solution (equivalent to 100 mg glucose g^−1^ dw pig manure) was added to samples for the SIR assay. The evolved CO_2_ was trapped in 0.02 M and 0.06 M NaOH (basal and SIR respectively) and then measured by titration with HCl to a phenolphthalein endpoint, after adding excess BaCl_2_.

We analyzed enzymes of the C cycle, such as β-glucosidase and cellulase, because they have been associated with litter mass loss and therefore with the turnover of carbon in a wide range of ecosystems [37]–[39]. Alkaline phosphatase is involved in the P cycle, hydrolysing organic P esters [40] to inorganic phosphorus, which then is available to plants. Protease activity arising from by depolymerization of dissolved organic nitrogen from N-containing compounds [41], is assumed to be a critical point in the N cycle [42] as polymers are not accessible to microorganisms [43]. Therefore, since these enzymes metabolize large organic polymers into smaller ones, analyzing them we can study decomposition rates of organic matter.

β-glucosidase activity was assessed by determination of the released *p*-nitrophenol, after the incubation of samples (1 g fresh weight) with *p*-nitrophenyl glucoside (0.025 M) for 1 hour at 37°C, in a Bio-Rad Microplate Reader at 400 nm [44]. Cellulase activity was estimated by determination of released reducing sugars, after incubation of samples (5 g fresh weight) with carboxymethyl cellulose sodium salt (0.7%) for 24 hours at 50°C, in a Bio-Rad Microplate Reader at 690 nm [45]. Protease activity was measured by determination of amino acids released, after incubation of samples (1 g fresh weight) with sodium caseinate (2%) for 2 hours at 50°C using Folin-Ciocalteu reagent, in a Bio-Rad Microplate Reader 550 at 700 nm [46]. Alkaline phosphatase activity was estimated by determination of *p*-nitrophenol (PNP) released, after incubation of samples (1 g fresh weight) with *p*-nitrophenyl phosphate (0.025 M) for 1 hour at 37°C, in a Bio-Rad Microplate Reader 550 at 400 nm [47].

### Statistical analyses

We calculated the strength and direction of indirect effects of earthworms (i.e. effect of inoculation) as the change in the variables in the presence of vermicompost (2.5 and 10%) relative to the control (no vermicompost), as (V_i_-V_c_)/V_c_, where V_i_ and V_c_ are the values of the variables of samples inoculated with vermicompost or controls. Negative values indicate that inoculation reduces the performance of the microbial community whereas positive values indicate an enhancement of microbial community functioning. In order to explore the effect of the different treatments applied (type of vermicompost, dose of inoculation and time), the data were analyzed by an ANOVA in which three factors were fixed: type of vermicompost (from *E. fetida*, *E. andrei* and *E. eugeniae*), dose of inoculation (2.5 and 10%) and time (15, 30 and 60 days). All analyses were performed with R [48].
